# The Naples prognostic score as a new predictive index of severe abdominal aortic calcification: a population-based study

**DOI:** 10.3389/fcvm.2025.1545927

**Published:** 2025-02-18

**Authors:** Qiang Tan, Jian Zhang, Yanrong Peng, Rui Yang, Yanbin Zhu, Xi Yong, Hongshun Yin, Jianghua Zheng

**Affiliations:** Vascular Surgery, Affiliated Hospital of North Sichuan Medical College, Nanchong Sichuan, China

**Keywords:** Naples prognostic score, abdominal aortic calcification, cardiovascular disease, NHANES, cross-sectional study

## Abstract

**Purpose:**

Abdominal aortic calcification (AAC) is related to inflammation and nutritional status. The Naples prognostic score (NPS) is an innovative biological marker capable of reflecting systemic inflammation and nutritional status. This research seeks to investigate the correlation of NPS with severe abdominal aortic calcification (SAAC).

**Methods:**

The research evaluated data obtained from the National Health and Nutrition Examination Survey (NHANES) conducted between 2013 and 2014. The variables were filtered utilizing the Least Absolute Shrinkage and Selection Operator (LASSO) regression. Weighted logistic regression models were employed to examine the association of NPS with SAAC. The predictive value of NPS for the risk of SAAC was assessed utilizing the receiver operating characteristic (ROC) curve. A subgroup analysis was conducted to assess the strength and reliability of the research findings.

**Results:**

The research encompassed 2,854 participants, among whom 303 (11.87%) exhibited SAAC. The outcomes of multivariate weighted logistic regression revealed that participants with a NPS of 3–4 points was positively correlated with SAAC in comparison to the control group [odds ratio (OR) = 2.07, 95% confidence interval (95%CI): 1.17–3.67]. The area under the curve (AUC) for predicting the risk of SAAC using NPS was 0.635. The subgroup analysis results indicated that there was no significant difference noted in the association of NPS with SAAC across various population subgroups.

**Conclusion:**

A positive association of NPS with SAAC has been observed in this research. This study offers valuable insights into the prevention and diagnosis of SAAC. Future longitudinal studies are warranted to confirm causative relationships and assess the role of NPS in clinical decision-making for SAAC.

## 1 Introduction

Vascular calcification (VC) describes the abnormal buildup of different minerals within the intima or inner layer of blood vessels, involving complex physiological and pathological processes, including lipid deposition, inflammatory reactions, disorders of calcium and phosphorus metabolism, and phenotypic changes in vascular smooth muscle cells (1–3). It is a sign of the development of atherosclerotic plaque (4) and is closely related to cardiovascular disease (CVD).

Abdominal aortic calcification (AAC) is a frequent presentation of VC. The occurrence rate and severity of AAC rise with advancing age and are correlated to traditional CV risk factors (5). Prior research has indicated that AAC serves as an independent predictor of CV events and death rate, with severe abdominal aortic calcification (SAAC) having a stronger predictive effect and impact. AAC has a closer correlation with the overall mortality rate of CVD in comparison to coronary artery calcification (6–8), and it is also related to the prognosis of vascular disease after surgery (9, 10). In addition, SAAC is a risk factor for symptomatic or even ruptured abdominal aortic aneurysms (11). A recent study also found that areas of the aorta with high levels of calcification are more likely to develop pseudoaneurysms or aortic ulcers (12). Therefore, in clinical practice, there is a pressing requirement for cost-effective, straightforward, and readily available markers for early detection and routine screening to prevent and alleviate AAC.

Malnutrition and inflammation are closely related to VC (13). Current research suggests that some inflammatory indicators are associated with the severity of AAC, involving systemic immune-inflammation index (SII) (14), pan-immune-inflammation value (PIV) (15), and monocyte-to-lymphocyte ratio (MLR) (16). Reduced expressions of serum albumin (SA) and total cholesterol (TC) are linked to CVD and peripheral vascular disease (PVD) (17, 18). Currently, limited research has been conducted to investigate the correlation of dietary inflammatory index with CVD. The Naples prognostic score (NPS) is a comprehensive index derived from SA, TC, lymphocyte to monocyte ratio (LMR), and neutrophil to lymphocyte ratio (NLR) proposed by Galizia et al. (19) It has been proven to be a novel prognostic indicator for various cancers. NPS is also recognized as an independent predictive factor of all-cause death rate in individuals with myocardial infarction and heart failure (20, 21), and is closely correlated to mortality and amputation rates after PAD revascularization (22).

It is important to highlight that there is currently a lack of research on the association of NPS with AAC. Hence, a cross-sectional analysis was undertaken utilizing data from the National Health and Nutrition Examination Survey (NHANES) spanning from 2013 to 2014. This study is novel in its exploration of the association between NPS and SAAC. While NPS has been previously utilized as a prognostic marker in various conditions, its role as a predictive index for SAAC has not been investigated. This analysis aims to bridge this gap, offering a novel biomarker for the early diagnosis and prevention of SAAC.

## 2 Methods

### 2.1 Data source and study population

**Figure 1:**
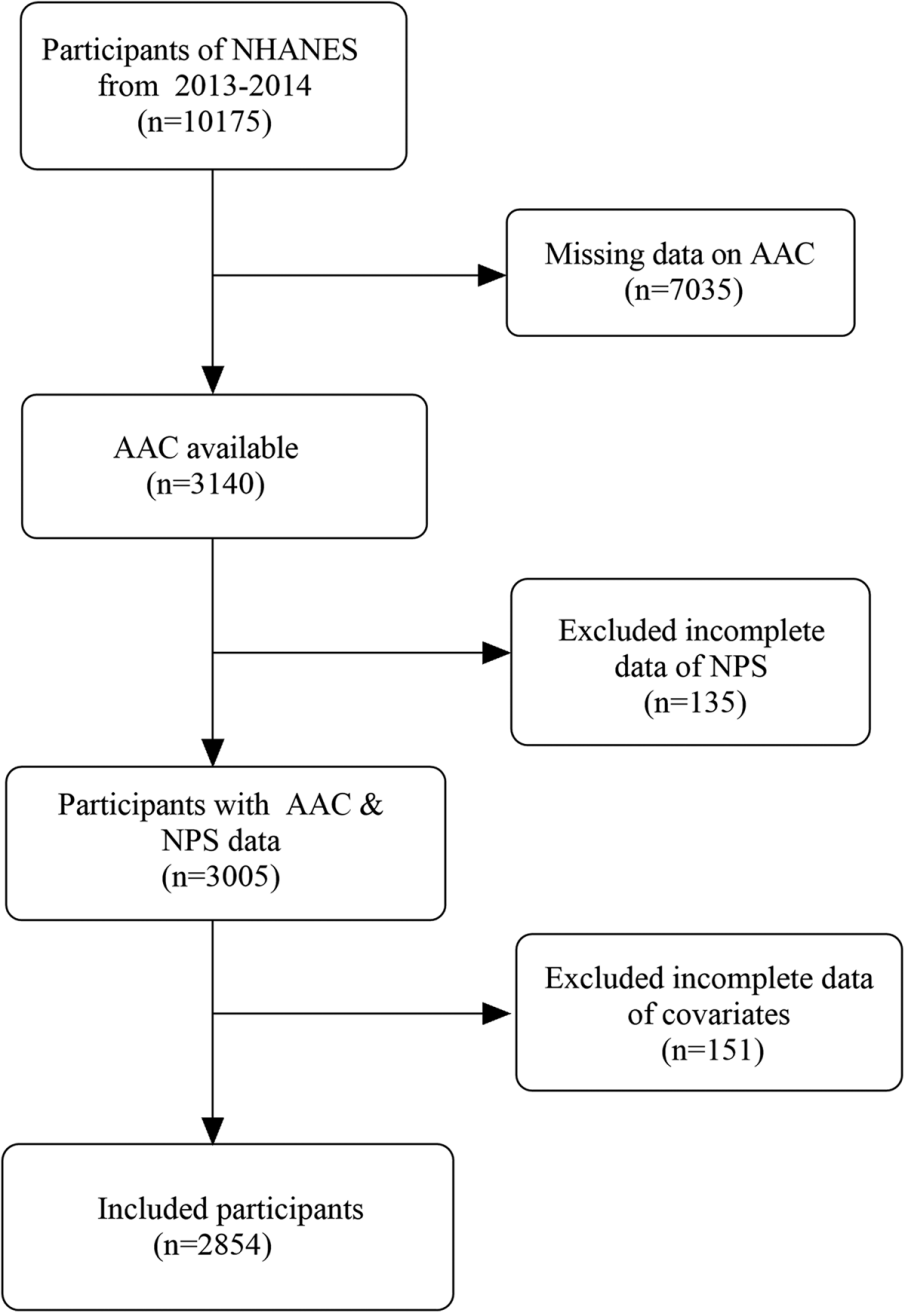
Flowchart of participants selection from NHANES. NHANES, National Health and Nutrition Examination Survey; AAC, abdominal aortic calcification; NPS, Naples prognostic score.

NHANES, administered by the National Center for Health Statistics (NCHS), is an ongoing, comprehensive cross-sectional survey designed to assess the health and nutritional condition of both children and adults across the United States (23). The NHANES 2013–2014 cohort containing AAC data was adopted. Altogether 10,175 individuals completed the survey, including participants aged 40 and above who underwent AAC evaluation. Altogether 3,005 participants with valid information on AAC and NPS were included in this study. Following the exclusion of 151 participants due to missing covariate data, incorporating smoking, waist circumference, body mass index (BMI), blood biochemical parameters, and other comorbidities, 2,854 participants were ultimately enrolled in this research. The detailed procedure of screening research data is depicted in Figure 1. All data were available at the official website (https://www.cdc.gov/nchs/nhanes). The research methodology has received endorsement from the NCHS Research Ethics Review Committee, with all participants duly submitting informed consent documentation (14).

### 2.2 SAAC assessment

The state of AAC was assessed through lateral scanning of the lumbar spine employing dual-energy x-ray absorptiometry (DXA) and quantified utilizing the Kauppila scoring system. The system, which spans from 0 to 24 points, partitions the anterior and posterior walls of the aorta into four segments aligned with the lumbar vertebrae L1–L4. Distinct scores (ranging from 0 to 6 points) were assigned to each region based on the proportion of calcification, with higher scores indicating more obvious calcification (24). The principal outcome variable under scrutiny in this study was SAAC. Based on previous relevant research, an AAC score of ≥6 was determined as SAAC (25–28). Comprehensive information regarding the assessment of AAC can be found at https://wwwn.cdc.gov/Nchs/Nhanes/2013-2014/DXXAAC_H.htm.

### 2.3 NPS assessment

The NPS was derived from a computation involving four key indicators: SA, TC, LMR, and NLR. Each of the following criteria was assigned a score of 1: SA level < 40 g/L, TC level ≤ 180 mg/dl, NLR > 2.96, and LMR ≤ 4.44. The cumulative score was determined by adding up the individual scores from these indicators (19). According to previous studies (29), patients were categorized into three cohorts according to their NPS value: Group 0 comprising individuals with a total score of 0, Group 1 with a total score ranging from 1 to 2, and Group 2 with a total score between 3 and 4.

### 2.4 Relevant covariates

The relevant covariates included demographic data, testing data, laboratory indicators, smoking status, and comorbidities. Demographic variables encompassed age, sex, race, and educational background; Testing data encompassed BMI (kg/m^2^) and waist circumference (cm); Laboratory indicators included urinary albumin to creatinine ratio (mg/g), serum calcium (mmol/L), serum phosphorus (mmol/L), blood uric acid (umol/L), hemoglobin (g/dl), erythrocyte distribution width (%), platelets, glycosylated hemoglobin (%), vitamin D (nmol/L), and vitamin B12 (pmol/L); Comorbidities included hypertension, diabetes, hypercholesterolemia, kidney stones, arthritis, gout, cancer and osteoporosis. A detailed description of these covariates is available at https://wwwn.cdc.gov/nchs/nhanes/continuousnhanes/default.aspx?BeginYear=2013.

### 2.5 Statistical analysis

Continuous variables' normality was assessed utilizing the Shapiro–Wilk test, revealing that none of the continuous variables in this research followed a normal distribution. Therefore, median and interquartile range (IQR) were employed to describe continuous variables, whereas counts and percentages were adopted for categorical variables. Non-normally distributed continuous variables were assessed utilizing the Mann–Whitney *U* test. Categorical variable comparisons were conducted employing either the chi-square test or Fisher's exact test.

Relevant variables were filtered utilizing the Least Absolute Shrinkage and Selection Operator (LASSO) regression technique. The association of NPS with SAAC was assessed utilizing a multivariate weighted logistic regression model, with the outcomes presented as odds ratio (OR) and 95% confidence intervals (CIs). Model 1 did not incorporate any adjusted covariates. Age, smoking, and BMI were adjusted in model 2. In Model 3, covariates such as hypertension, diabetes, tumor and hypercholesterolemia were adjusted on the basis of model 2. The ROC curve was utilized to assess the predictive capacity of NPS regarding the risk of SAAC. The area under the ROC curve (AUC) was calculated along with 95% confidence intervals (CI), and thresholds for sensitivity and specificity were also determined. AUC values ≥ 0.5 were considered indicative of diagnostic utility, with statistical significance set at *P* < 0.05. The stability of the association between NPS and SAAC was evaluated by subgroup analysis of gender, age, educational level, smoking, diabetes, hypertension, tumor, hypercholesterolemia, kidney stones, arthritis, gout, and osteoporosis. In addition, an interaction test was performed to assess the diversity in correlations among subgroups. All statistical analyses were conducted utilizing R software (V4.3). A two-sided *P*-value of less than 0.05 was deemed the threshold for statistical significance.

## 3 Results

### 3.1 Baseline features

Table 1 displays the baseline attributes of the participants. Altogether 2,854 individuals were finally enrolled in this study, including 1,375 (48%) men and 1,479 women (52%), and 1,231 (38%) patients ≥60 years old. Race was categorized into Mexican Americans (6.9%), non-Hispanic whites (71%), non-Hispanic blacks (9.7%), and other races (12%). About 303 participants had SAAC (11.87%). The median serum values of NLR, LMR, SA, and TC were 2.06 [IQR: 1.55, 2.71], 3.50 [IQR: 2.78, 4.50], 43 [IQR: 41, 44] g/L, and 193 [IQR: 166, 221] mg/dl, respectively. Patients were grouped according to NPS, with 485 participants divided into Group 0 (0 points), 1,961 participants divided into Group 1 (1–2 points), and 408 participants divided into Group 2 (3–4 points). Compared with the participants without severe AAC, individuals in the SAAC group exhibited advanced age, elevated NPS values, a higher prevalence of smoking, and were more prone to conditions such as hypertension, diabetes, hypercholesterolemia, arthritis, cancer, and osteoporosis, had lower BMI, hemoglobin, and platelets, and a higher value of urinary albumin to creatinine ratio, blood uric acid, red blood cell distribution width, glycosylated hemoglobin, vitamin D, and vitamin B12.

**Table 1:** Baseline characteristics of the study population by categories of SAAC.

Characteristic	All (*n* = 2,854)	Without SAAC (*n* = 2,551)	With SAAC (*n* = 303)	*P*-value
Male, *n* (%)	1,375 (48%)	1,228 (49%)	147 (45%)	0.493
Age, *n* (%)				
<60	1,623 (62%)	1,571 (67%)	52 (18%)	<0.001
≥60	1,231 (38%)	980 (33%)	251 (82%)	
Race, *n* (%)				
Mexican American	377 (6.9%)	349 (7.2%)	28 (4.5%)	0.020
Non-Hispanic White	1,268 (71%)	1,078 (71%)	190 (79%)	
Non-Hispanic Black	535 (9.7%)	498 (10.0%)	37 (6.4%)	
Other Race	674 (12%)	626 (12%)	48 (9.8%)	
Education categories, *n* (%)				
<High school diploma	638 (15%)	563 (14%)	75 (20%)	0.047
High school diploma	643 (22%)	565 (21%)	78 (26%)	
>high school diploma	1,573 (63%)	1,423 (64%)	150 (54%)	
Smoking, *n* (%)				
Never	1,550 (54%)	1,426 (56%)	124 (40%)	0.002
Former	781 (28%)	663 (27%)	118 (41%)	
Current	523 (17%)	462 (17%)	61 (20%)	
BMI (kg/m^2^)	27.9 (24.7, 31.8)	28.0 (24.6, 32.0)	26.8 (24.7, 29.9)	0.015
WC (cm)	100 (90, 109)	100 (90, 109)	98 (92, 107)	0.360
Hypertension, *n* (%)	1,329 (43%)	1,112 (41%)	217 (69%)	<0.001
Diabetes, *n* (%)	486 (14%)	395 (12%)	91 (27%)	<0.001
High cholesterol, *n* (%)	1,344 (49%)	1,154 (47%)	190 (65%)	<0.001
Kidney stones, *n* (%)	312 (12%)	278 (12%)	34 (10%)	0.480
Arthritis, *n* (%)	966 (36%)	831 (34%)	135 (48%)	<0.001
Gout, *n* (%)	145 (5.6%)	121 (5.5%)	24 (6.2%)	0.643
Cancer, *n* (%)	359 (15%)	284 (13%)	75 (30%)	<0.001
Osteoporosis, *n* (%)	233 (8.1%)	184 (7.3%)	49 (16%)	0.002
ACR (mg/g)	7 (5.13)	7 (5.12)	12 (7.26)	<0.001
Serum calcium (mmol/L)	2.35 (2.30, 2.43)	2.35 (2.30, 2.43)	2.38 (2.30, 2.43)	0.090
Serum phosphorus (mmol/L)	1.23 (1.10, 1.36)	1.23 (1.10, 1.36)	1.26 (1.13, 1.36)	0.073
SUA (umol/L)	315 (262, 375)	315 (262, 369)	327 (280, 387)	0.008
Hemoglobin (g/dl)	14.10 (13.30, 15.00)	14.20 (13.30, 15.10)	13.90 (12.70, 14.70)	0.033
RDW (%)	13.40 (13.00, 14.00)	13.40 (13.00, 13.90)	13.60 (13.10, 14.20)	<0.001
Platelet count	225 (192, 265)	225 (192, 267)	217 (178, 256)	<0.001
Glycohemoglobin	5.50 (5.30, 5.90)	5.50 (5.30, 5.80)	5.90 (5.50, 6.40)	<0.001
Total 25-hydroxyvitamin D (nmol/L)	72 (56, 91)	71 (55, 90)	80 (64, 96)	0.002
Vitamin B12 (pmol/L)	381 (284, 530)	378 (283, 525)	420 (302, 596)	0.031
NLR	2.06 (1.55, 2.71)	2.05 (1.54, 2.68)	2.33 (1.65, 3.14)	0.003
LMR	3.50 (2.78, 4.50)	3.50 (2.80, 4.50)	3.00 (2.33, 4.00)	<0.001
SA (g/L)	43 (41, 44)	43 (41, 45)	42 (40, 44)	0.097
TC (mg/dl)	193 (166, 221)	195 (167, 222)	181 (161, 210)	0.011
NPS group				
0	485 (15%)	462 (16%)	23 (8.0%)	0.003
1–2	1,961 (71%)	1,759 (72%)	202 (69%)	
3–4	408 (13%)	330 (12%)	78 (23%)	

SAAC, severe abdominal aortic calcification; BMI, body mass index; WC, waist circumference; ACR, albumin creatinine ratio; SUA, serum uric acid; RDW, red cell distribution width; NLR, neutrophil to lymphocyte ratio; LMR, lymphocyte-to-monocyte ratio; SA, serum albumin; TC, total cholesterol; NPS, Naples prognostic score.

Continuous variables without a normal distribution are presented as medians [interquartile ranges]. Categorical variables were shown as numbers and percentages (%).

### 3.2 LASSO regression evaluation for variable filtering

**Figure 2:**
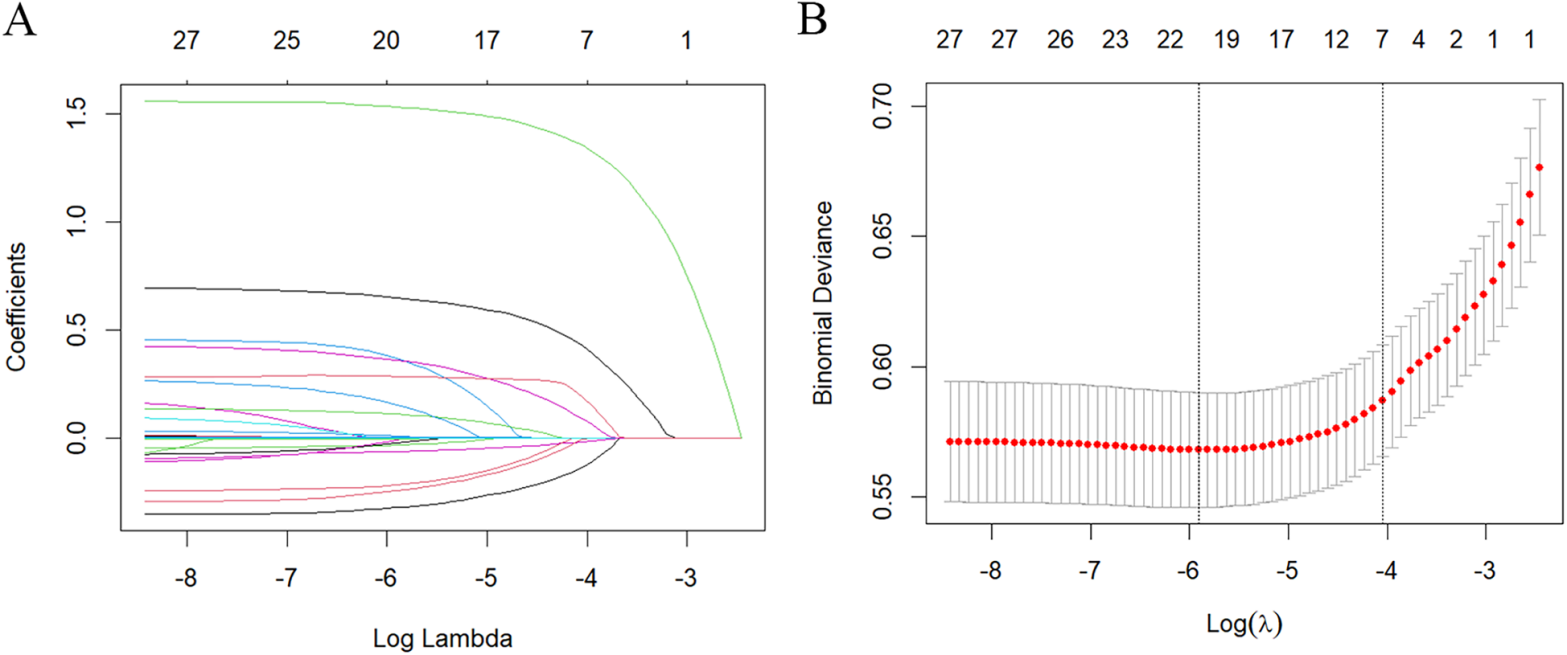
Characteristics screening for SAAC prediction by lasso regression. **(A)** Regression coefficient profile diagram. Each curve represents the change trajectory of each characteristic coefficient. **(B)** Cross-validation curve of Lasso regression. Each red dot represents the Mean Squared Error (MSE) for each value of *λ*. The ordinate shows the coefficient values, while the upper abscissa indicates the number of non-zero coefficients in the model, and the lower abscissa displays the logarithmic value of the regularization parameter *λ*. The dashed line on the left indicates the minimum *λ* value, while the dashed line on the right indicates the *λ* value with one standard error. For SAAC prediction, we used the lambda.1se for variable selection.

To identify confounders that affect the incidence rate of SAAC, in addition to NPS and its indicators (NLR, LMR, SA and TC), LASSO regression evaluation was conducted on the covariates, and a 10-fold cross-validation was performed. The “lambda. 1se” was employed for variable selection, resulting in the final selection of seven feature variables: age, BMI, smoking, diabetes, hypertension, cancer, and hypercholesterolemia (Figure 2).

### 3.3 Relationship between NPS and SAAC

The relationship between NPS (both as a continuous and a categorical variable) and SAAC is detailed in Table 2. Following adjustment for potential confounders (Model 3), NPS exhibited a statistically significant positive correlation with SAAC when it was a continuous variable (OR = 1.24, 95% CI = 1.08, 1.43, *P* = 0.009). When NPS was treated as a categorical variable, NPS = 0 was established as the reference group. As for the unadjusted model, compared with Group 1, Group 2 was positively correlated with SAAC (OR = 3.59, 95% CI = 2.04, 6.32); Even after additional adjustments for age and BMI in model 2, Group 2 still showed a significant positive correlation with SAAC compared to Group 1 (OR = 2.21, 95% CI = 1.32, 3.71); Upon accounting for all potential confounders including age, BMI, smoking, diabetes, hypertension, cancer and hypercholesterolemia in model 3, this correlation still had statistical significance (OR = 2.07, 95% CI = 1.17, 3.67).

**Table 2:** Association between NPS and SAAC.

	Model 1		Model 2		Model 3	
	OR (95% CI)	*P*-value	OR (95% CI)	*P*-value	OR (95% CI)	*P*-value
NPS*	1.51 (1.30, 1.74)	<0.001	1.33 (1.17, 1.50)	<0.001	1.24 (1.08, 1.43)	0.009
NPS**						
0	–	–	–	–	–	–
1–2	1.92 (0.98, 3.79)	0.058	1.55 (0.78, 3.08)	0.2	1.70 (0.78, 3.68)	0.14
3–4	3.59 (2.04, 6.32)	<0.001	2.21 (1.32, 3.71)	0.006	2.07 (1.17, 3.67)	0.022

NPS, Naples prognostic score; SAAC, severe abdominal aortic calcification; BMI, body mass index; NPS*, NPS as a continuous variable; NPS**, NPS as a categorical variable; OR, odds ratio; CI, confidence interval.

Model 1: no covariate was adjusted.

Model 2: Adjust for age, BMI, smoke.

Model 3: Adjust for age, BMI, smoking, hypertension, diabetes, high cholesterol, cancer.

### 3.4 The predictive performance of NPS for SAAC

**Figure 3:**
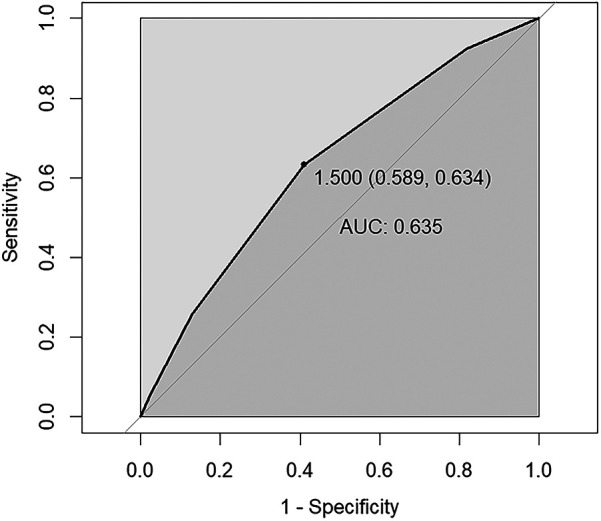
ROC curve assesses the predictive value of NPS for SAAC. The area under the curve (AUC) is 0.635 (95% CI = 0.603–0.666), *P* < 0.001. The specificity and sensitivity of the model were 0.60 and 0.63 respectively. NPS, Naples prognostic score; SAAC, severe abdominal aortic calcification.

The ROC curve was employed to test the predictive efficacy of NPS for SAAC, with the findings depicted in Figure 3. The optimal critical value for NPS prediction of SAAC was 1.5, with specificity and sensitivity of 0.60 and 0.63, respectively. The area under the curve (AUC) was 0.635, with a 95% confidence interval of 0.603–0.666, and *P* < 0.001, indicating that NPS had a modest but statistically significant predictive effect on SAAC.

### 3.5 Subgroup analysis

**Figure 4:**
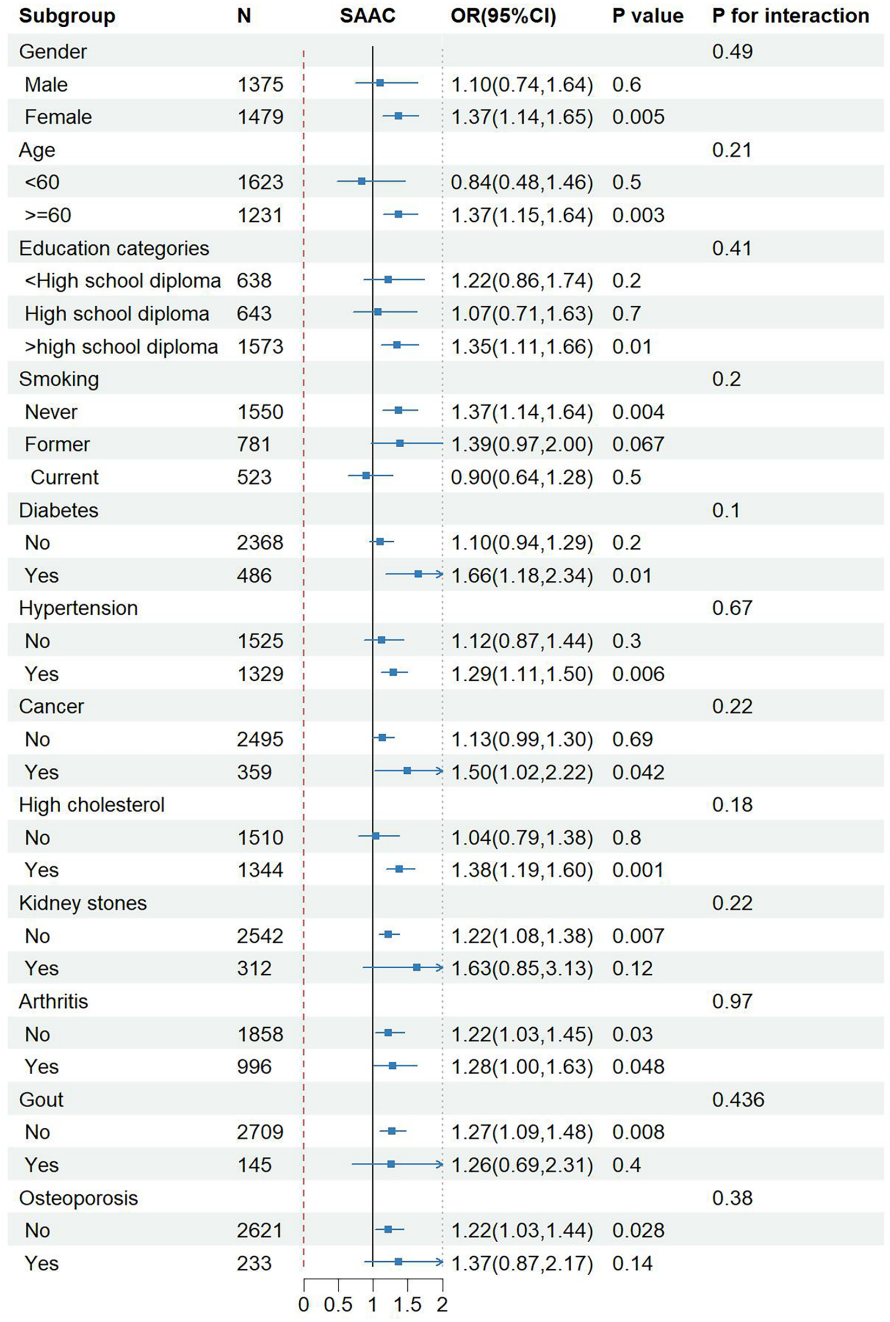
Subgroup analysis for the association between NPS and SAAC. Adjusted for age, BMI, smoking, diabetes, hypertension, cancer, high cholesterol. NPS, Naples prognostic score; SAAC, severe abdominal aortic calcification; BMI, body mass index; OR, odds ratio; CI, confidence interval.

To assess the reliability of the link between NPS and SAAC within specific populations and find out potential different populations, subgroup analysis and interaction tests were carried out based on sex, age, educational level, smoking, diabetes, hypertension, cancer, hypercholesterolemia, kidney stones, arthritis, gout and osteoporosis. Notably, NPS exhibited a more pronounced association with the risk of SAAC in women, people ≥60 years old, people with high school education or above, people with hypertension and diabetes, people with hypercholesterolemia and tumors, and people without arthritis, gout and osteoporosis. However, the *P*-value from the interaction test exceeded 0.05 across all subgroups, without statistical significance, indicating that the association between NPS and SAAC did not depend on gender, age, educational level, smoking, diabetes, hypertension, cancer, and hypercholesterolemia. The subgroup analysis findings suggested that no notable variations were observed in this positive correlation among different population stratifications (Figure 4).

## 4 Discussion

The study utilized extensive and representative data sourced from the NHANES database, encompassing the U.S. population from 2013 to 2014. To date, our study has explored for the first time the relationship between NPS and SAAC. The research results indicate a positive correlation between NPS and SAAC. After adjusting for relevant confounders, the incidence of SAAC increased by 24% for every 1 point increase in NPS. In comparison to the control group, the incidence of SAAC was higher when NPS was 3–4 points [OR: 2.07 (1.17–3.67)]. The subgroup analysis showed that the results were robust. Due to the cost-effectiveness, convenience, and easy access of NPS, it can provide potential application value for clinical diagnosis of SAAC. These findings hold promise for future investigations into the association of NPS with AAC.

VC usually occurs in coronary arteries, lower extremity arteries, aorta and its branches. AAC is a marker of advanced atherosclerosis, which can more directly assess vascular injury and more accurately reflect the potential atherosclerotic burden. It stands out as a robust predictor of CV adverse events and death, surpassing the Framingham risk score (6, 7, 30). In addition, AAC has the potential to modify the local mechanical and hemodynamic properties of blood vessels (31), increase the peak wall stress of abdominal aortic aneurysm (AAA), reduce its biomechanical stability, increase the risk of aortic ulcer, aneurysm rupture, and dissection progression, and impacts the extended-term survival following endovascular repair of abdominal aneurysm (EVAR) (10–12). Given the potential adverse effects of AAC, early and timely diagnosis and corresponding measures to prevent the progression of AAC are particularly important.

Nutritional status and inflammation levels are correlated with atherosclerotic CVD (32, 33). Systemic inflammation could result in alterations in neutrophil, lymphocyte, monocyte, and platelet levels. Recent cross-sectional research from NHANES has demonstrated the correlation between several inflammatory biomarkers and SAAC (14–16). Nevertheless, there is a scarcity of research investigating the connection among malnutrition, inflammation, and AAC (13). Increased levels of serum high-sensitivity C-reactive protein (hs-CRP) and reduced SA are autonomous indicators of AAC progression across hemodialysis patients. NPS is a novel comprehensive indicator based on four indicators including SA, TC, LMR, and NLR, which reflects the nutritional and systemic inflammatory condition of participants. Following its initial introduction as a biomarker, research on NPS has mainly focused on its diagnostic and prognostic value for tumors. For example, NPS has been demonstrated to serve as an autonomous risk factor for the extended-term survival of breast cancer patients receiving neoadjuvant chemotherapy (34), hepatocellular carcinoma patients receiving hepatectomy (35), metastatic colorectal cancer and (36) lung cancer patients (37). In addition, NPS can predict the all-cause death rate within 30 days in individuals diagnosed with acute pulmonary embolism (38) and is correlated to the prognosis of CVD (myocardial infarction, heart failure) and PAD after revascularization (20–22, 39). An increase in NPS levels suggests the progression and poor prognosis of various diseases, which is consistent with our research findings.

Neutrophils mediate inflammatory reactions through a variety of biochemical mechanisms, such as promoting platelet aggregation and adhesion, secreting inflammatory mediators, and releasing arachidonic acid derivatives, cytotoxic oxygen free radicals and proteolytic enzymes (including myeloperoxidase, elastase and acid phosphatase) to make plaque more vulnerable, which is positively related to atherosclerosis burden and ischemic status (40). Inflammation can also increase lymphocyte apoptosis, and a reduced lymphocyte count suggests a compromised immune response, linked to more severe CVD outcomes (22). Monocytes are recruited and develop into macrophages in the early stage of atherosclerosis. Macrophages and activated lymphocytes exhibit high expression of SPP1 (secreted phosphoprotein-1 or osteopontin) within atherosclerotic plaques, thereby fostering the development of fat stripes and plaques while contributing to the calcification procedure in atherosclerotic plaques (41). Therefore, a decrease in LMR and an increase in NLR may suggest more severe inflammatory responses and VC.

Nutritional status can alter the metabolic state of tissues and play a crucial role in disease progression, which is associated with inflammation and CVD (42, 43). A recent study (44) has found that hs-CRP and low-density lipoprotein cholesterol can predict the likelihood of CV events and death, and the risk of atherosclerosis can be reduced through active combination of lipid-lowering and anti-inflammatory therapy. SA serves various physiological roles, such as antioxidant, anti-inflammatory, anticoagulant, and anti-platelet aggregation activities. It regulates immune response, prevents endothelial cell apoptosis, dilates blood vessels, and safeguards tissues from ischemic injury. Reduced serum albumin levels have been linked to ischemic heart disease, heart failure, atrial fibrillation, stroke, PAD, and venous thromboembolism, and are an independent risk factor (45–48). Cheng et al. (17) found that SA levels equal to or greater than 3.75 g/dl at admission had a correlation with a reduction in long-term CV mortality, encompassing deaths from ischemic heart disease, congestive heart disease, and stroke. Lower SA levels can serve as predictors of unfavorable cardiac events (myocardial infarction, percutaneous coronary intervention and coronary artery bypass surgery, and mortality) in individuals with advanced atherosclerosis (47, 49). In addition, PAD patients with lower levels of SA and TC have an increased long-term mortality rate after receiving endovascular treatment (18).

A single inflammatory or nutritional biomarker may not be sufficient to evaluate SAAC. NPS integrates multiple biomarkers that mirror the nutritional and immune condition of patients, making it a more comprehensive indicator than a single inflammatory or nutritional marker. The decrease in SA, TC, and NLR levels, as well as the elevation in LMR levels, are correlated to an increase in NPS, indicating more severe inflammatory reactions and poorer nutritional conditions in patients. This could explain why elevated NPS is positively correlated with SAAC.

There are several significant strengths in this study. First of all, to the best of our understanding, this study is pioneering in offering insights into the association of NPS with SAAC. In addition, we have incorporated a population-based study that is nationally representative to bolster the generalizability and inclusiveness of our research outcomes. Moreover, we have performed LASSO regression analysis to exclude potential confounders from the results. Finally, the subgroup analysis has shown that the results are robust. Furthermore, the limitations of this study must be acknowledged. First of all, given the cross-sectional analysis design, establishing a causal association of NPS with SAAC is not feasible. Future longitudinal studies and randomized controlled trials are imperative to validate this association. Secondly, owing to constraints within the database, we could not encompass data on all covariates influencing AAC and dietary inflammation levels. Third, only data on the population of the United States are collected in our database, which may only apply to a limited number of people. Therefore, further prospective and multi-center studies are essential to validate this finding.

## 5 Conclusion

To sum up, our study has found that an increase in NPS is positively correlated with the incidence of SAAC. The NPS may hold potential value in clinical practice for the identification and prevention of SAAC. Further exploration is needed in the future to confirm the association between the two and elucidate its potential mechanisms.

## Data Availability

The datasets presented in this study can be found in online repositories. The names of the repository/repositories and accession number(s) can be found below: https://www.cdc.gov/nchs/nhanes.

## References

[1] Villa-Bellosta R. Vascular calcification: key roles of phosphate and pyrophosphate. Int J Mol Sci. (2021) 22(24):13536. 10.3390/ijms222413536 34948333 PMC8708352

[2] Johnson RC Leopold JA Loscalzo J. Vascular calcification: pathobiological mechanisms and clinical implications. Circ Res. (2006) 99(10):1044–59. 10.1161/01.RES.0000249379.55535.21 17095733

[3] Quaglino D Boraldi F Lofaro FD. The biology of vascular calcification. Int Rev Cell Mol Biol. (2020) 354:261–353. 10.1016/bs.ircmb.2020.02.007 32475476

[4] Nakahara T Strauss HW. From inflammation to calcification in atherosclerosis. Eur J Nucl Med Mol Imaging. (2017) 44(5):858–60. 10.1007/s00259-016-3608-x 28062896

[5] Bartstra JW Mali W Spiering W de Jong PA. Abdominal aortic calcification: from ancient friend to modern foe. Eur J Prev Cardiol. (2021) 28(12):1386–91. 10.1177/2047487320919895 34647579

[6] Bastos Goncalves F Voute MT Hoeks SE Chonchol MB Boersma EE Stolker RJ Calcification of the abdominal aorta as an independent predictor of cardiovascular events: a meta-analysis. Heart. (2012) 98(13):988–94. 10.1136/heartjnl-2011-301464 22668866

[7] Criqui MH Denenberg JO McClelland RL Allison MA Ix JH Guerci A Abdominal aortic calcium, coronary artery calcium, and cardiovascular morbidity and mortality in the Multi-Ethnic Study of Atherosclerosis. Arterioscler Thromb Vasc Biol. (2014) 34(7):1574–9. 10.1161/ATVBAHA.114.303268 24812323 PMC4153597

[8] Wilson PW Kauppila LI O'Donnell CJ Kiel DP Hannan M Polak JM Abdominal aortic calcific deposits are an important predictor of vascular morbidity and mortality. Circulation. (2001) 103(11):1529–34. 10.1161/01.CIR.103.11.1529 11257080

[9] Harbaugh CM Terjimanian MN Lee JS Alawieh AZ Kowalsky DB Tishberg LM Abdominal aortic calcification and surgical outcomes in patients with no known cardiovascular risk factors. Ann Surg. (2013) 257(4):774–81. 10.1097/SLA.0b013e31826ddd5f 23001086

[10] TerBush MJ Rasheed K Young ZZ Ellis JL Glocker RJ Doyle AJ Aortoiliac calcification correlates with 5-year survival after abdominal aortic aneurysm repair. J Vasc Surg. (2019) 69(3):774–82. 10.1016/j.jvs.2018.05.242 30292612

[11] Buijs RV Willems TP Tio RA Boersma HH Tielliu IF Slart RH Calcification as a risk factor for rupture of abdominal aortic aneurysm. Eur J Vasc Endovasc Surg. (2013) 46(5):542–8. 10.1016/j.ejvs.2013.09.006 24091093

[12] Li S Kan H Liu Z Zeng R Shao J Chen Y Aortic calcification correlates with pseudoaneurysm or penetrating aortic ulcer of different etiologies. Sci Rep. (2024) 14(1):25. 10.1038/s41598-023-49429-y 38167947 PMC10761832

[13] Choi SR Lee YK Cho AJ Park HC Han CH Choi MJ Malnutrition, inflammation, progression of vascular calcification and survival: inter-relationships in hemodialysis patients. PLoS One. (2019) 14(5):e0216415. 10.1371/journal.pone.0216415 31048884 PMC6497382

[14] Xie R Liu X Wu H Liu M Zhang Y. Associations between systemic immune-inflammation index and abdominal aortic calcification: results of a nationwide survey. Nutr Metab Cardiovasc Dis. (2023) 33(7):1437–43. 10.1016/j.numecd.2023.04.015 37156667

[15] Jin C Li X Luo Y Zhang C Zuo D. Associations between pan-immune-inflammation value and abdominal aortic calcification: a cross-sectional study. Front Immunol. (2024) 15:1370516. 10.3389/fimmu.2024.1370516 38605946 PMC11007162

[16] Ban TH Choi BS Yoon SA Kim Y Jin K Kim GH Clinical significance of neutrophil-to-lymphocyte ratio on the risk of abdominal aortic calcification and decreased bone mineral density in patients with end-stage kidney disease. PLoS One. (2023) 18(10):e0286612. 10.1371/journal.pone.0286612 37878613 PMC10599515

[17] Cheng CW Lee CW Chien SC Yeh HI Chen CY. Serum albumin was associated with a long term cardiovascular mortality among elderly patients with stable coronary artery disease. Acta Cardiol Sin. (2024) 40(1):87–96. 10.6515/ACS.202401_40(1).20230825A 38264075 PMC10801420

[18] Mizobuchi K Jujo K Minami Y Ishida I Nakao M Hagiwara N. The baseline nutritional status predicts long-term mortality in patients undergoing endovascular therapy. Nutrients. (2019) 11(8):1745. 10.3390/nu11081745 31362417 PMC6722841

[19] Galizia G Lieto E Auricchio A Cardella F Mabilia A Podzemny V Naples prognostic score, based on nutritional and inflammatory status, is an independent predictor of long-term outcome in patients undergoing surgery for colorectal cancer. Dis Colon Rectum. (2017) 60(12):1273–84. 10.1097/DCR.0000000000000961 29112563

[20] Erdogan A Genc O Ozkan E Goksu MM Ibisoglu E Bilen MN Impact of Naples prognostic score at admission on in-hospital and follow-up outcomes among patients with ST-segment elevation myocardial infarction. Angiology. (2023) 74(10):970–80. 10.1177/00033197231151559 36625023

[21] Kilic O Suygun H Mustu M Ozpamuk Karadeniz F Ozer SF Senol H Is the Naples prognostic score useful for predicting heart failure mortality. Kardiologiia. (2023) 63(3):61–5. 10.18087/cardio.2023.3.n2328 37061862

[22] Artac I Karakayali M Omar T Ilis D Arslan A Hakan Sahin M Predictive value of the Naples prognostic score on long-term outcomes in patients with peripheral artery disease revascularized via percutaneous intervention. Ann Vasc Surg. (2024) 102:121–32. 10.1016/j.avsg.2023.11.028 38307231

[23] Zipf G Chiappa M Porter KS Ostchega Y Lewis BG Dostal J. National health and nutrition examination survey: plan and operations, 1999–2010. Vital Health Stat 1. (2013) (56):1–37. 25078429

[24] Schousboe JT Lewis JR Kiel DP. Abdominal aortic calcification on dual-energy x-ray absorptiometry: methods of assessment and clinical significance. Bone. (2017) 104:91–100. 10.1016/j.bone.2017.01.025 28119178

[25] Xue X Li C Chen D. A cross-sectional study investigating the relationship between urinary albumin creatinine ratio and abdominal aortic calcification in adults. Front Cardiovasc Med. (2024) 11:1352921. 10.3389/fcvm.2024.1352921 38500760 PMC10944970

[26] Smith C Sim M Dalla Via J Gebre AK Zhu K Lim WH Extent of abdominal aortic calcification is associated with incident rapid weight loss over 5 years: the perth longitudinal study of ageing women. Arterioscler Thromb Vasc Biol. (2024) 44(2):e54–64. 10.1161/ATVBAHA.123.320118 38095109 PMC10832333

[27] Lewis JR Schousboe JT Lim WH Wong G Wilson KE Zhu K Long-term atherosclerotic vascular disease risk and prognosis in elderly women with abdominal aortic calcification on lateral spine images captured during bone density testing: a prospective study. J Bone Miner Res. (2018) 33(6):1001–10. 10.1002/jbmr.3405 29443425 PMC6415911

[28] Bondonno NP Lewis JR Prince RL Lim WH Wong G Schousboe JT Fruit intake and abdominal aortic calcification in elderly women: a prospective cohort study. Nutrients. (2016) 8(3):159. 10.3390/nu8030159 26978394 PMC4808887

[29] Zhu N Lin S Yu H Liu F Huang W Cao C. Naples Prognostic score as a novel prognostic prediction indicator in adult asthma patients: a population-based study. World Allergy Organ J. (2023) 16(10):100825. 10.1016/j.waojou.2023.100825 37954399 PMC10632111

[30] O'Connor SD Graffy PM Zea R Pickhardt PJ. Does nonenhanced CT-based quantification of abdominal aortic calcification outperform the framingham risk score in predicting cardiovascular events in asymptomatic adults? Radiology. (2019) 290(1):108–15. 10.1148/radiol.2018180562 30277443

[31] Li ZY U-King-Im J Tang TY Soh E See TC Gillard JH. Impact of calcification and intraluminal thrombus on the computed wall stresses of abdominal aortic aneurysm. J Vasc Surg. (2008) 47(5):928–35. 10.1016/j.jvs.2008.01.006 18372154

[32] Violi F Pastori D Pignatelli P Carnevale R. Nutrition, thrombosis, and cardiovascular disease. Circ Res. (2020) 126(10):1415–42. 10.1161/CIRCRESAHA.120.315892 32379574

[33] Kong P Cui ZY Huang XF Zhang DD Guo RJ Han M. Inflammation and atherosclerosis: signaling pathways and therapeutic intervention. Signal Transduct Target Ther. (2022) 7(1):131. 10.1038/s41392-022-00955-7 35459215 PMC9033871

[34] Xiu Y Jiang C Huang Q Yu X Qiao K Wu D Naples score: a novel prognostic biomarker for breast cancer patients undergoing neoadjuvant chemotherapy. J Cancer Res Clin Oncol. (2023) 149(17):16097–110. 10.1007/s00432-023-05366-x 37698677 PMC11797591

[35] Xie YM Lu W Cheng J Dai M Liu SY Wang DD Naples prognostic score is an independent prognostic factor in patients undergoing hepatectomy for hepatocellular carcinoma. J Hepatocell Carcinoma. (2023) 10:1423–33. 10.2147/JHC.S414789 37691971 PMC10488664

[36] Park SH Woo HS Hong IK Park EJ. Impact of postoperative Naples prognostic score to predict survival in patients with stage II–III colorectal cancer. Cancers. (2023) 15(20):5098. 10.3390/cancers15205098 37894465 PMC10605496

[37] Liu J Wang Z Liu G Liu Z Lu H Ji S. Assessment of Naples prognostic score in predicting survival for small cell lung cancer patients treated with chemoradiotherapy. Ann Med. (2023) 55(2):2242254. 10.1080/07853890.2023.2242254 37552770 PMC10411310

[38] Zhu N Lin S Cao C. A novel prognostic prediction indicator in patients with acute pulmonary embolism: Naples prognostic score. Thromb J. (2023) 21(1):114. 10.1186/s12959-023-00554-8 37932805 PMC10629175

[39] Saylik F Cinar T Selcuk M Akbulut T Hayiroglu MI Tanboga IH. Evaluation of Naples score for long-term mortality in patients with ST-segment elevation myocardial infarction undergoing primary percutaneous coronary intervention. Angiology. (2024) 75(8):725–33. 10.1177/00033197231170982 37058422

[40] Spark JI Sarveswaran J Blest N Charalabidis P Asthana S. An elevated neutrophil-lymphocyte ratio independently predicts mortality in chronic critical limb ischemia. J Vasc Surg. (2010) 52(3):632–6. 10.1016/j.jvs.2010.03.067 20573475

[41] Zhao Y Huang Z Gao L Ma H Chang R. Osteopontin/SPP1: a potential mediator between immune cells and vascular calcification. Front Immunol. (2024) 15:1395596. 10.3389/fimmu.2024.1395596 38919629 PMC11196619

[42] Di Giosia P Stamerra CA Giorgini P Jamialahamdi T Butler AE Sahebkar A. The role of nutrition in inflammaging. Ageing Res Rev. (2022) 77:101596. 10.1016/j.arr.2022.101596 35219904

[43] Kunimura A Ishii H Uetani T Aoki T Harada K Hirayama K Impact of nutritional assessment and body mass index on cardiovascular outcomes in patients with stable coronary artery disease. Int J Cardiol. (2017) 230:653–8. 10.1016/j.ijcard.2017.01.008 28077227

[44] Ridker PM Bhatt DL Pradhan AD Glynn RJ MacFadyen JG Nissen SE Inflammation and cholesterol as predictors of cardiovascular events among patients receiving statin therapy: a collaborative analysis of three randomised trials. Lancet. (2023) 401(10384):1293–301. 10.1016/S0140-6736(23)00215-5 36893777

[45] Yan P Tang Q Wu Y Wan Q Zhang Z Xu Y Serum albumin was negatively associated with diabetic peripheral neuropathy in Chinese population: a cross-sectional study. Diabetol Metab Syndr. (2021) 13(1):100. 10.1186/s13098-021-00718-4 34526116 PMC8444578

[46] Arques S. Human serum albumin in cardiovascular diseases. Eur J Intern Med. (2018) 52:8–12. 10.1016/j.ejim.2018.04.014 29680174

[47] Manolis AA Manolis TA Melita H Mikhailidis DP Manolis AS. Low serum albumin: a neglected predictor in patients with cardiovascular disease. Eur J Intern Med. (2022) 102:24–39. 10.1016/j.ejim.2022.05.004 35537999

[48] Folsom AR Lutsey PL Heckbert SR Cushman M. Serum albumin and risk of venous thromboembolism. Thromb Haemost. (2010) 104(1):100–4. 10.1160/TH09-12-0856 20390234 PMC2902783

[49] Schillinger M Exner M Mlekusch W Amighi J Sabeti S Schlager O Serum albumin predicts cardiac adverse events in patients with advanced atherosclerosis—interrelation with traditional cardiovascular risk factors. Thromb Haemost. (2004) 91(3):610–8. 10.1160/TH03-08-0504 14983239

